# Fibroblast growth factor 23 expression in human calcified vascular tissues

**DOI:** 10.18632/aging.102297

**Published:** 2019-09-22

**Authors:** Javier Donate-Correa, Ernesto Martín-Núñez, Carolina Hernández-Carballo, Carla Ferri, Víctor G. Tagua, Alejandro Delgado-Molinos, Ángel López-Castillo, Sergio Rodríguez-Ramos, Purificación Cerro-López, Victoria Castro López-Tarruella, Raquel Felipe-García, Miguel A. Arévalo-Gomez, Nayra Pérez-Delgado, Carmen Mora-Fernández, Juan F. Navarro-González

**Affiliations:** 1 Research Unit, University Hospital Nuestra Señora de Candelaria (UHNSC), Santa Cruz de Tenerife, Spain; 2 Doctoral and Graduate School, University of La Laguna, San Cristóbal de La Laguna, Tenerife, Spain; 3 Vascular Surgery Service, UHNSC, Santa Cruz de Tenerife, Spain; 4 Transplant Coordination, UHNSC, Santa Cruz de Tenerife, Spain; 5 Pathology Service, UHNSC, Santa Cruz de Tenerife, Spain; 6 Human Anatomy and Histology Department, University of Salamanca, Salamanca, Spain; 7 Clinical Analysis Service, UHNSC, Santa Cruz de Tenerife, Spain; 8 Nephrology Service, UHNSC, Santa Cruz de Tenerife, Spain; 9 Biomedical Technologies Institute, University of La Laguna, Tenerife, Spain

**Keywords:** vascular calcification, FGF23, Klotho

## Abstract

Vascular calcification is a major risk for cardiovascular disease and implies the transformation of smooth muscle cells to an osteoblastic phenotype as a consequence of dysregulation of calcium and phosphate metabolism. Fibroblast growth factor (FGF) 23 is the most potent phosphate regulator. Observational studies suggest that high levels of FGF23 are related to cardiovascular morbidity and mortality. In this work, we determined the levels of both the intact and the carboxi-terminal fragments of circulating FGF23 in 133 patients with established cardiovascular disease, the expression of FGF23, its receptors 1 and 3, and its co-receptor Klotho in vascular fragments of aorta, carotid and femoral in 43 out of this group of patients, and in a control group of 20 organ donors. Patients with atherosclerosis and vascular calcification presented increased levels of FGF23 respect to the control group. Vascular immunoreactivity for FGF23 was also significantly increased in patients with vascular calcification as compared to patients without calcification and to controls. Finally, gene expression of *FGF23* and *RUNX2* were also higher and directly related in vascular samples with calcification. Conversely, expression of *Klotho* was reduced in patients with cardiovascular disease when comparing to controls. In conclusion, our findings link the calcification of the vascular tissue with the expression of FGF23 in the vessels and with the elevation of circulating levels this hormone.

## INTRODUCTION

Vascular calcification (VC) constitutes a major risk factor for cardiovascular (CV) morbidity and mortality and involves a complex regulated process of biomineralization that resembles osteogenesis [1]. This process is mainly driven by the vascular smooth muscle cells (VSMCs), and includes the transformation of these cells into an osteoblastic phenotype [2].

Chronic kidney disease (CKD) is a major risk factor for CV disease (CVD) and is a clinical scenario closely related to the development of VC. In addition to the traditional CV risk factors, subjects with CKD are also exposed to other non-traditional factors predisposing for this pathology [3]. Fibroblast growth factor (FGF) 23 is the most potent phosphatonin [4]. This is an osteocyte-derived hormone produced in response to phosphate levels which, in combination with its cofactor Klotho, reduces the reabsorption of phosphate and the synthesis of active vitamin D in the kidneys [5]. In patients with CKD, FGF23 concentrations increase with declining renal function and reach extremely high levels in end-stage renal disease. Clinical epidemiological studies have shown that FGF23 strongly predicts mortality in patients with CKD independently of other risk factors [6, 7]. These results suggest that FGF23 may causally be related to the high mortality observed in CKD patients and, importantly, that may exert direct effects on CV system besides its function as a phosphaturic hormone.

FGF23 binds to its cognate receptors (FGFRs), which are activated in the presence of the co-receptor Klotho [8]. Our group and others described the expression of FGFR and Klotho in the human vascular wall [9, 10], allowing to speculate that vascular tissue may be an objective for the actions of FGF23. Moreover, the synthesis of FGF23 by extra-osseous calcified tissues and its contribution to CVD is an intriguing question not adequately studied. Only two previous works have explored the expression of FGF23 in calcified tissues [11, 12], although solely coronary arteries and carotid atheroma plaques were analyzed. Moreover, the relationships of vascular FGF23 gene and protein expression levels with soluble FGF23 concentration and with the expression of Klotho and FGFRs in the vessels have not been previously established.

In this work, we investigated in a group of patients with established CVD the expression of FGF23, the receptors FGFR1 and 3, and the co-receptor Klotho in different large vascular territories (aorta, carotid or femoral arteries), and their relationships with circulating levels of FGF23 and with the presence of VC.

## RESULTS

### Soluble FGF23 and VC

**Figure 1:**
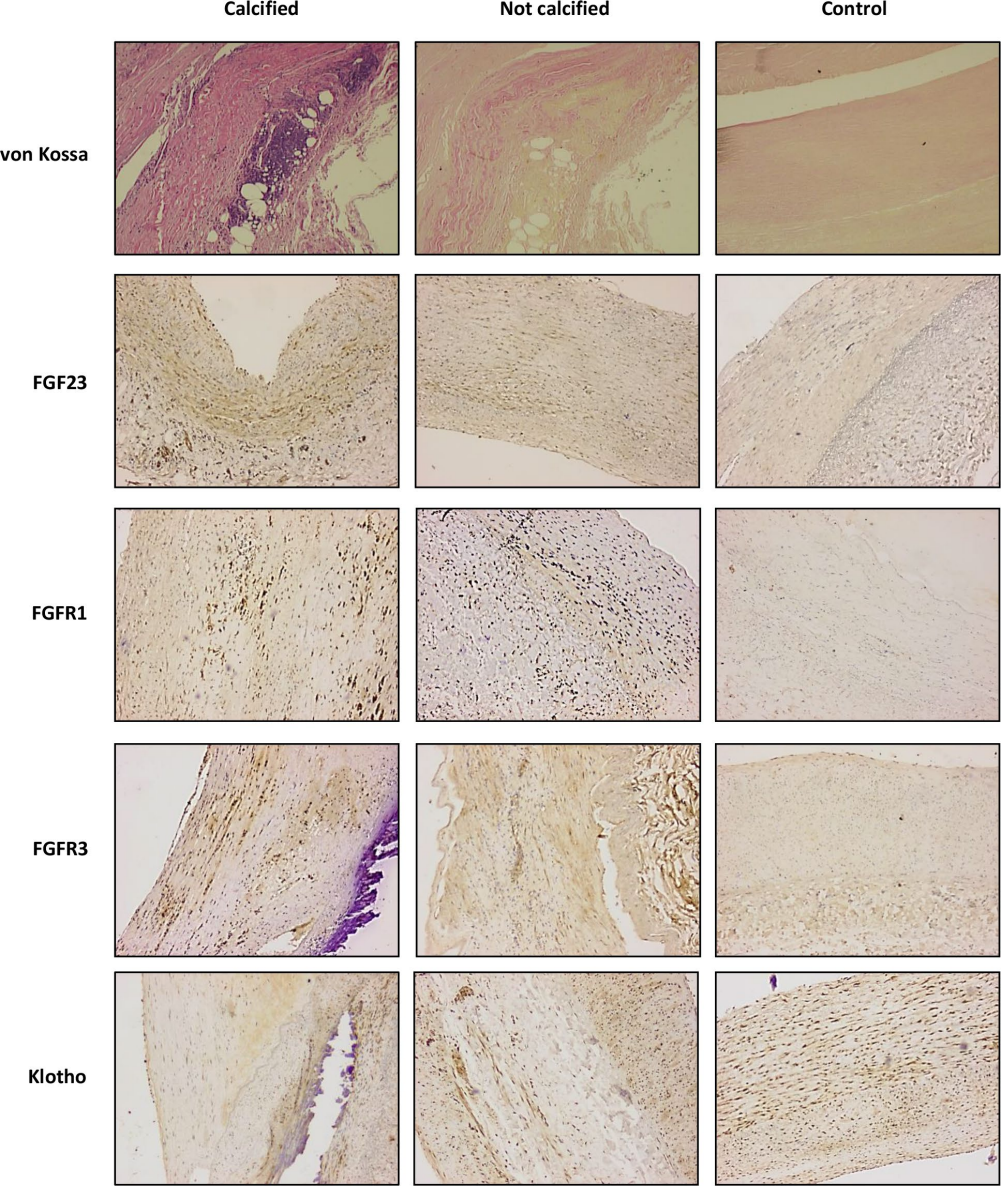
**von Kossa and immunohistochemichal staining for FGF23, FGFR1, FGFR3 and Klotho, in sections of calcified and not calcified arteries of vascular surgery patients and in sections of control donors (magnification 4x).**

The clinical, biochemical and demographic characteristics of the study population are presented in Table 1. From 180 potential subjects in the vascular surgery group who were initially considered, 47 were excluded according to exclusion criteria and 133 patients were finally included. The mean age was 69.2±9.8 years, with 75% of males and 45% diabetics. The mean eGFR was 78.8±24 mL/min/1.73 m^2^. VC was diagnosed in 62 patients (46.6%) by image procedures, including X-rays, ultrasonography, echocardiography, and/or computed tomography. The presence of arterial calcification was confirmed by von Kossa staining after vascular tissue retrieval (Figure 1).

**Table 1:** Clinical characteristics and biochemical assessments of patients included in the study.

**Variable**	**Overall**	**Calcified tissue**	**Not calcified tissue**	***P* value**
***Characteristics***				
N (%)	133	62 (46.6)	71 (53.4)	
Age (years)	68.4±8.5	69.2±7.5	67.7±9.2	NS
Male gender, n (%)	100 (75)	46 (74)	54 (76)	NS
Systolic BP (mm Hg)	129±20	131±20	126±19	NS
Diastolic BP (mm Hg)	68.4±12.3	69±13.8	68±10.8	NS
BMI (kg/m^2^)	28.2±4.1	28±4	28.4±4.2	NS
***Comorbidities***				
Hypertension, n (%)	111 (83)	55 (89)	56 (79)	NS
Diabetes mellitus, n (%)	62 (47)	34 (55)	28 (40)	NS
Alcoholism, n (%)	56 (42)	29 (47)	27 (38)	NS
Smoking habits, n (%)	93 (70)	45 (73)	48 (67)	NS
Dyslipidaemia, n (%)	27 (20)	11 (18)	16 (23)	NS
***Laboratory data***				
Total Cholesterol (mg/dL)	168±45	160.8±53	174.3±41	NS
HDL (mg/dL)	44.2±13.7	45.2±15.4	43.4±12	NS
LDL (mg/dL)	92.8±36	87.5±34	97.6±37	NS
Triglycerides, mg/dL	148.2±91	143.5±101	152.3±81	NS
HbA1c, %	6.3±1.4	6.1±1.1	6.5±1.5	NS
eGFR, mL/min/1.73 m^2^	76.4±21	73.1±23	79.3.5±20	NS
Albumin (g/L)	3.8±0.6	3.8±0.6	3.8±0.6	NS
Albuminuria	54±64	56±64	51±65	NS
Calcium (mg/dL)	9.1±0.5	9.1±0.5	9±0.6	NS
Phosphorous (mg/dL)	3.6±0.5	3.6±0.5	3.6±0.6	NS
TAP (mU/mL)	64 (43-81)	70.5 (47-91.5)	61 (32-75)	0.07
hs-CRP (mg/dL)	4.7 (1.9-11)	5.3 (2.1-13)	4.5 (1.6-11)	NS
iFGF23, pg/mL	20.1 (12.4-26.1)	22.9 (15.6-29.8)	15.6 (9.5-22.2)	<0.01
cFGF23, RU/mL*	29.1 (22.3-34)	29.9 (26.4-42.9)	21 (12.1-30.7)	<0.01

BP, blood pressure; BMI, body mass index; HDL, high density lipoprotein; LDL, low density lipoprotein; HbA1c, hemoglobin A1c; eGFR, estimated glomerular filtration rate; TAP, total alkaline phosphatase; hs-CRP, high sensitivity C-reactive protein; PTH, parathyroid hormone; FGF23, fibroblast growth factor 23; NS, not significant. P value reflects differences between calcified and non-calcified tissue. *Serum levels of 66 patients (37 with and 29 without VC).

**Figure 2:**
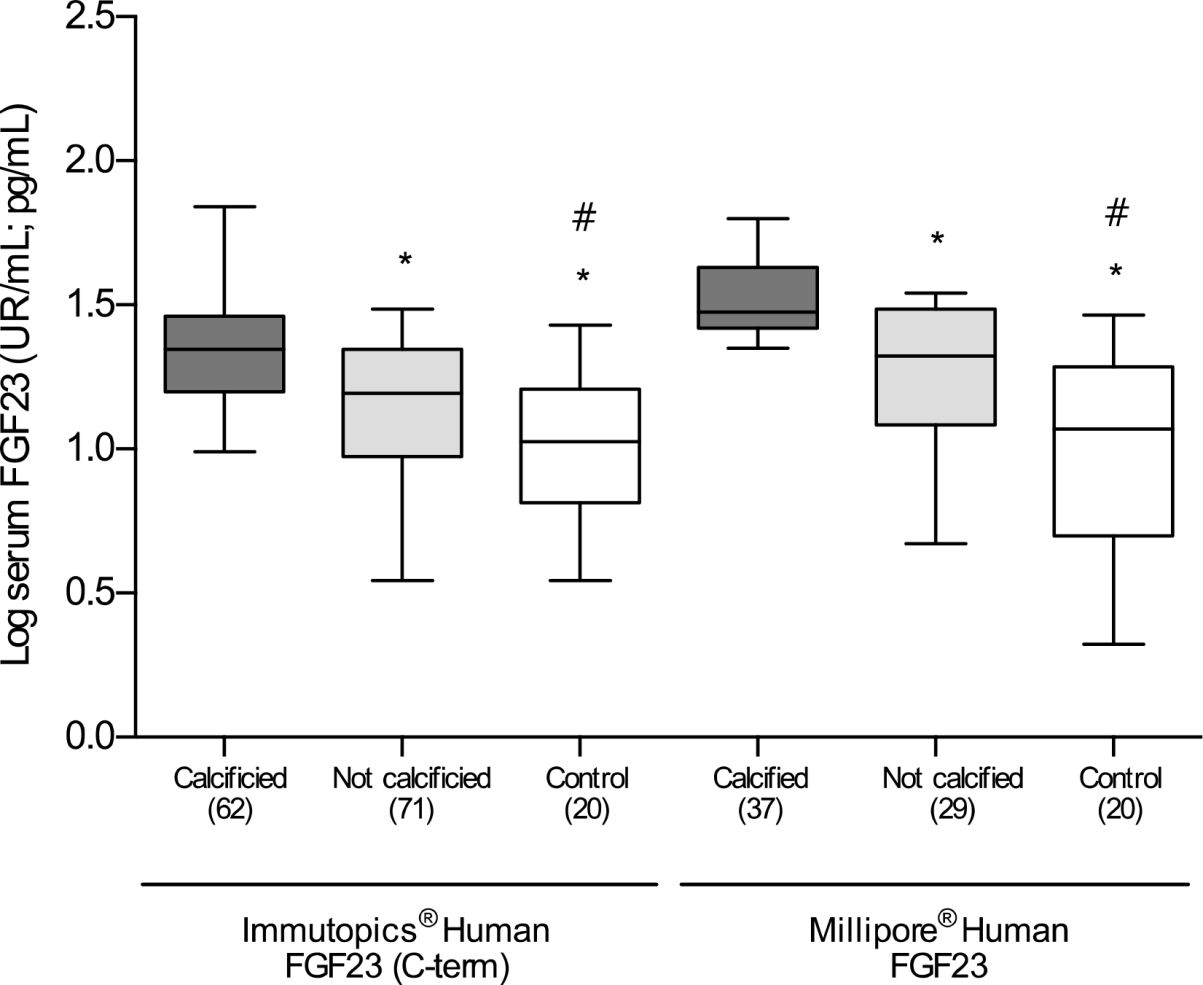
**Differences in the log-transformed blood levels of FGF23, determined by two different ELISA kits, among calcified, not calcified and control groups.** *P<0.01 vs. calcified group; #P<0.01 vs. non-calcified group.

Comparison of patients classified according to the presence or absence of VC showed no differences regarding comorbidities, including hypertension, alcoholism, smoking habit, or dyslipidemia. The incidence of diabetes mellitus in the group of patients with VC was higher (55%) than in those without VC (40%) although this difference was not statistically significant. Similarly, no differences were observed in general laboratory measurements that included, among others, total cholesterol, HDL, LDL, calcium, phosphorous, high sensitivity C-reactive protein (hs-PCR) and eGFR. Total alkaline phosphatase (TAP) levels were higher in patients with VC, with a difference that almost reached statistical significance (P=0.07). However, and interestingly, the levels of both the intact form and the C-terminal fragments of FGF23 were significantly higher in patients with VC [iFGF23: 22.7 (11.1-56.4) *vs.* 15.2 (3.3-41.1) pg/mL, P<0.01; cFGF23: 27.2 (9.3-91.9) *vs.* 21.9 (10.2-57.9) RU/mL, P<0.01]. These differences represent a median percent increment of 49.3% and 24.2% respect to patients without VC, respectively (Table 1 and Figure 2).

As compared with the overall group of patients with established vascular disease, the serum concentrations of the two soluble forms of FGF23 were statistically lower in the subjects included in the control group [iFGF23: 10.1 (6.8-23) vs. 18.6 (9.8-45.3) pg/mL; cFGF23: 18.8 (3.6-31) vs. 23.6 (9.8-71.5) RU/mL; P<0.01 for both] (Figure 2). Patients with vascular disease without VC had a median percent increase in the serum concentrations of cFGF23 and iFGF23 of 17.1% and 50.5% in comparison to control subjects, respectively. Importantly, these differences were clearly higher in patients with VC, who showed a median percent increase in the soluble levels of cFGF23 and iFGF23 of 44.6% and 124.7%, respectively.

When analyzed the results obtained with both ELISA kits, we observed that there was a direct and significant correlation between them (r=0.63 P<0.0001), although the differences among the groups were higher with determining the iFGF23.

The characteristics of patients stratified by tertiles of soluble FGF23 are shown in Table 2. There were no differences concerning the prevalence of associated comorbid conditions except for diabetes mellitus, whose prevalence was significantly greater in subjects with higher soluble FGF23 concentrations (tertiles 2 and 3) (P<0.01). Regarding the differences in the levels of biochemical parameters including TAP, calcium and phosphorous, only albuminuria presented higher levels in the group of patients included in tertil 3 (P<0.05). The eGFR was significantly lower in subjects with FGF23 within the second and third tertiles (P<0.05). The percentage of patients with an eGFR below 60 mL/min/1.73 m^2^ increased with FGF23 tertiles (P<0.01 for iFGF23 and P<0.001 for cFGF23 determinations). Soluble FGF23 determined with both ELISA kits showed an inverse and significant correlation with eGFR (iFGF23: r=-0.383, P<0.0001; cFGF23: r=-0.413, P<0.0001).

**Table 2:** Clinical characteristics and general biochemical assessments stratified by tertiles of soluble iFGF-23 (pg/mL) and cFGF-23 (RU/mL) levels.

**Variable**	**Tertile 1 (<12 pg/mL / <23 RU/mL)**	**Tertile 2 (12-23 pg/mL / 21-47 RU/mL)**	**Tertile 3 (>23 pg/mL / >47 RU/mL)**	***P* value for trend**
***Characteristics***				
N (%)	44 (33.1) / 22 (16.5)	45 (33.8) / 22 (16.5)	44 (33.1) / 22 (16.5)	
Age (years)	67.1±9.1 / 70±9	67.6±11 / 69.1±13.3	69.5±9.8 / 68.3±10.1	NS / NS
Male gender (%)	34 (77.2) / 18 (81.8)	35 (77.7) / 17 (77.3)	31 (70.5) / 17 (77.3)	NS / NS
Systolic BP (mm Hg)	128.3±20.1 / 121.7±15.1	131.5±19.9 / 131.1±18.3	126.2±20 / 125±22.5	NS / NS
Diastolic BP (mm Hg)	68.7±10.3 / 65.3±11.3	69.5±13.5 / 68.5±8.4	67.1±12.8 / 64.9±12	NS / NS
BMI (kg/m^2^)	27.5±2.9 / 29.2±3.7	27.9±4.6 / 27.4±3.2	29.2±4.4 / 30.6±4.4	NS / NS
***Comorbidities***				
Hypertension (%)	33 (75) / 18 (81.8)	39 (88.8) / 15 (68.2)	39 (88.6) / 19 (86.4)	NS / NS
Diabetes mellitus (%)	17 (38.6) / 3 (13.6)	19 (42.2) / 8 (36.4)	23 (52.3) / 13 (59)	<0.01 / <0.01
Alcoholism (%)	20 (45.4) / 10 (45.4)	20 (44.4) / 8 (36.4)	16 (36.3) / 9 (40.9)	NS / NS
Smoking habits (%)	36 (81.8) / 17 (77.2)	33 (73.3) / 18 (81.8)	34(77.2) / 16 (72.7)	NS / NS
Dyslipidaemia (%)	9 (20.5) / 4 (18.1)	9 (20) / 5 (22.7)	10 (22.7) / 4 (18.1)	NS / NS
***Laboratory data***				
Total Cholesterol (mg/dL)	159.6±46.3 / 158.9±40.2	158.2±44.9 / 173.2±40.9	166.4±43.3 / 153.2±35.8	NS / NS
HDL (mg/dL)	44.4±12.6 / 41.1±14.8	44.6±15.9 / 51.8±19.6	43.7±12.5 / 43±11.1	NS / NS
LDL (mg/dL)	98.7±44.2 / 84.6±36.5	81.8±34.2 / 91.5±38	96.9±33.4 / 83.2±30.3	NS / NS
Triglycerides (mg/Dl)	156.9±125.6 / 147.8±80.3	158.2±87.8 / 125.7±55.2	129.2±37 / 137.2±56.9	NS / NS
HbA1c (%)	6.3±1.4 / 6.5±1.7	6.2±1.4 / 6.1±1.2	6.4±1.3 / 6.5±1.3	NS / NS
Albuminuria	22.1 (4.9-70.7) / 31 (5.3-132)	22 (0.6-63.2) / 28.2 (4.8-69.1)	56.5 (13.8-83) / 55.4 (25-88.4)	<0.05 / <0.05
Calcium (mg/dL)	9.1±0.6 / 9.2±0.5	8.9±0.6 / 8.7±0.7	9.1±0.4 / 8.9±0.6	NS / NS
Phosphorous (mg/dL)	3.5±0.5 / 3.6±0.5	3.6±0.5 / 3.5±0.4	3.7±0.5 / 3.7±0.6	NS / NS
TAP (mU/mL)	66 (52.2-79.2) / 63 (52.7-74.2)	56 (30.5-85.5) / 80.5 (56.5-101)	66 (32-80.2) / 64.5 (23.5-86.8)	NS / NS
hs-CRP (mg/dL)	4.1 (0.9-11) / 4.4 (0.4-13.1)	5.6 (2.1-12.9) / 5.2 (2.1-12.9)	4.6 (2.1-10.9) / 4.6 (0.9-6.3)	NS / NS
***Kidney function stage***				
eGFR (mL/ min/1.73 m^2^)	87.3±20.5 / 81±22.4	77.8±21.9 / 78.9±21.1	67.5±25.4 / 72.5±30.8	<0.05 / <0.05
eGFR < 60 mL/ min/1.73 m^2^ (%)	5 (11.3) / 3 (13.6)	12 (26.6) / 4 (18.2)	18 (40.9) / 8 (36)	<0.01 / <0.001

BP, blood pressure; BMI, body mass index; HDL, high density lipoprotein; LDL, low density lipoprotein; HbA1c, hemoglobin A1c; eGFR, estimated glomerular filtration rate; TAP, total alkaline phosphatase; hs-CRP, high sensitivity C-reactive protein; FGF23, fibroblast growth factor 23; NS, not significant.

### Histochemical and immunohistochemical analysis

For histochemical and immunohistochemical determinations, serial sections of different arterial territories were obtained from 43 surgical patients: 22 from patients with calcified arteries (4 aorta, 7 carotid and 11 femoral) and 21 from patients without VC (2 aorta, 8 carotid and 11 femoral). There were no significant differences in age (70±10 *vs.* 68.4±11 years), mean eGFR (87±11 *vs.* 91±9 mL/min/1.73 m2), sex (80% *vs.* 75% male) or prevalence of HT (78% *vs.* 81%), diabetes (46% *vs.* 51%) and smoking habits (61% *vs*. 56%). For comparative purposes, arterial samples were obtained from 18 control subjects (6 aorta and 12 femoral). The von Kossa staining confirmed the presence of calcific deposits in all the 22 sections obtained from patients diagnosed with VC. On the contrary, in the samples drawn from patients without clinical diagnosis of VC, as well as in control subjects, the von Kossa stain was negative (Figure 1).

**Figure 3:**
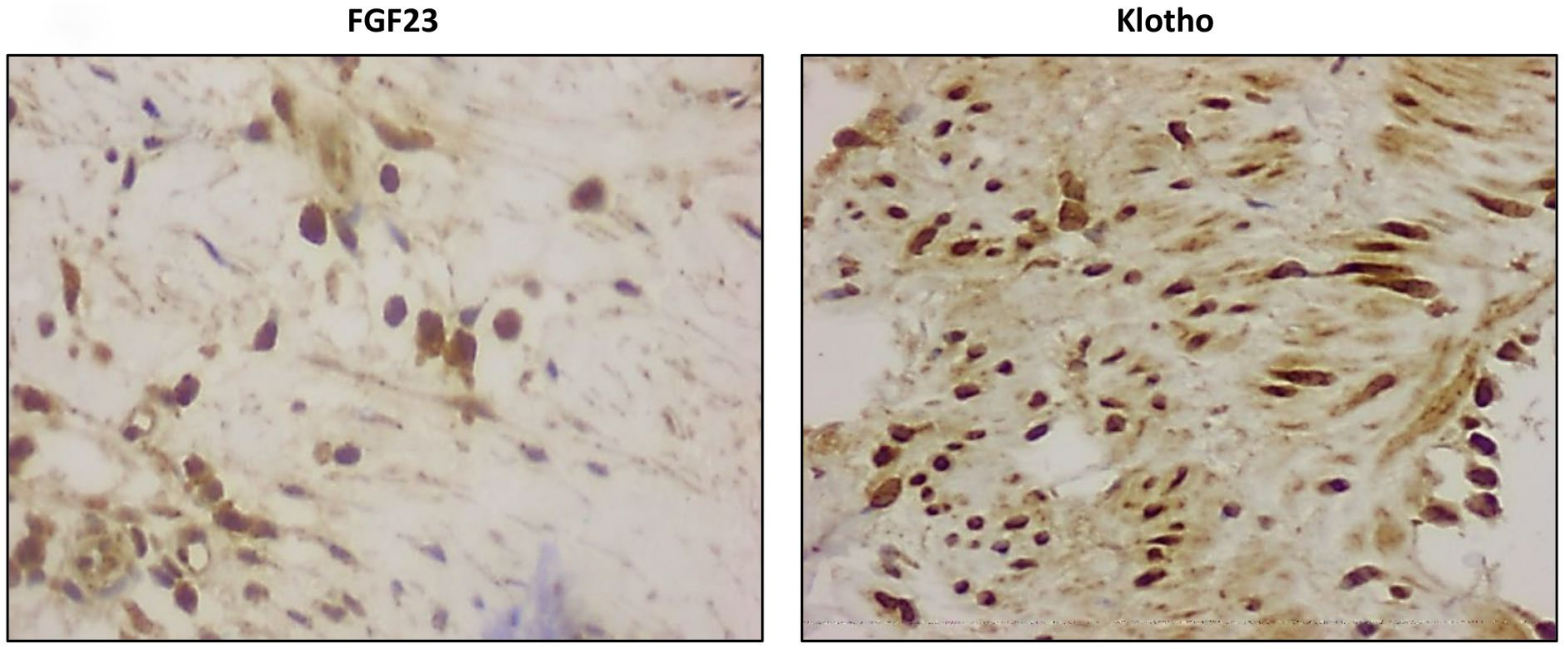
**Detection of intracellular FGF23 and Klotho expression (magnification x20).**

**Figure 4:**
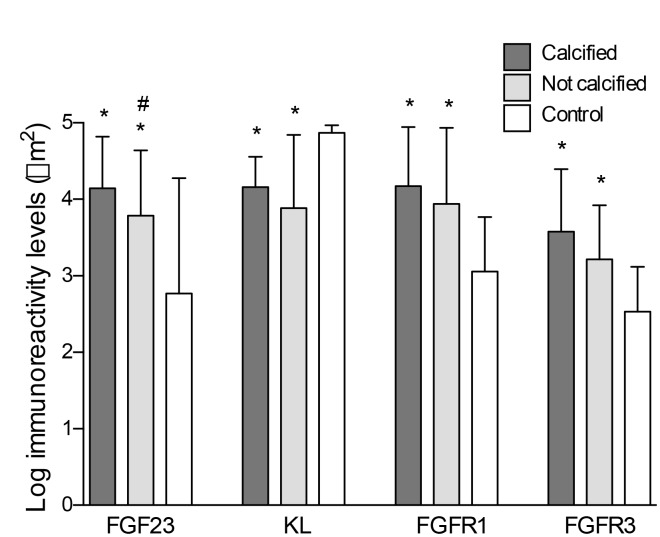
**Differences in the log-transformed immunoreactivity levels of FGF23, Klotho, FGFR1 and FGFR3 attending to the presence of calcification.** *P<0.01 vs. control group; #P<0.05 vs. non-calcified group.

FGF23 immunoreactivity was present in 18 out of the 22 (81.8%) samples with positive staining for calcification and in 12 of the 21 (57.1%) samples recovered from patients without vascular calcium deposits, whereas only 4 of the 18 controls (22.2%) presented positivity for FGF23 immunoreactivity. Klotho, FGFR1and FGFR3 were detected in all samples studied (Figure 1). In the samples that presented positive immunoreactivity for FGF23 and Klotho, the signal for these proteins was detected both intra- and extracellularly (Figure 3). Mean immunoreactivity levels for FGF23 were significantly increased in vascular sections of patients with VC as compared to sections from patients without calcification (P<0.05) and controls (P<0.01) (Figure 4). Interestingly, when comparing to the control group, Klotho immunoreactivity levels were reduced in patients with clinical atherosclerotic artery disease, independently of the presence or absence of VC (P<0.01). Finally, FGFR1 and FGFR3 signals were higher in the group of surgery patients than in controls (P<0.01).

The group of patients with histological study were also stratified by tertiles of soluble iFGF23 and cFGF23 levels. Image analysis revealed that immunoreactivity for FGF23 was statistically higher in the upper tertiles of soluble FGF23 (P<0.05). Conversely, Klotho signal was significantly reduced in those tertiles (P<0.05). No differences were observed in the immunoreactivity levels for FGFR1 and FGFR3 (Table 3).

**Table 3:** Immunoreactivity quantification and gene expression study stratified by tertiles of soluble iFGF23 (pg/mL) and cFGF23 (RU/mL) levels.

**Variable**	**Tertile 1 (<15 pg/mL / <24 RU/mL)**	**Tertile 2 (15-25 pg/mL / 24-46 RU/mL)**	**Tertile 3 (>25 pg/mL / >46 RU/mL)**	***P* value for trend**
**N (%)**	22 (34.4) / 21 (32.8)	21 (32.8) / 22 (34.4)	21 (32.8) / 22 (34.4)	
***Log Immunoreactivity levels (μm^2^)***				
FGF23	3.5±0.3 / 3.8±0.3	4.1±0.3 / 4.5±0.3	4.8±0.3 / 4.9±0.3	<0.05 / <0.05
FGFR1	4.5±0.5 / 4.6±0.3	4.4±.3 / 4.6±0.3	4.2± 0.4 / 4.5±0.3	NS / NS
FGFR3	3.5±0.4 / 3.4±0.7	3.5±0.4 / 3.4±0.9	3.6±0.3 / 3.5±0.8	NS / NS
Klotho	4.6±0.5 / 4.7±0.2	4.1±0.4 / 4.3±0.4	3.6±0.4 / 3.8±0.4	<0.05 / <0.05
***Log mRNA vascular levels (a.u.)***				
FGF23	5.1±2.1 / 4.8±3.3	6.1±2.3 / 6.5 ±0.3	7.2±3.6 / 4.9±0.3	<0.05 / <0.05
Klotho	4.3±0.5 / 4.5±0.3	4.1±.3 / 4.2±0.3	4± 1.4 / 3.9±0.3	NS / NS
***Kidney function stage***				
eGFR (mL/ min/1.73 m^2^)	85.3±31.6 / 84±24.1	74.5±26.2 / 77.7±26.5	68.3±15.6 / 70.7±25.6	<0.05 / <0.05

FGF23, fibroblast growth factor 23; FGFR, fibroblast growth factor receptor; a.u., arbitrary units; eGFR, estimated glomerular filtration rate; NS, not significant.

### Vascular gene expression

**Figure 5:**
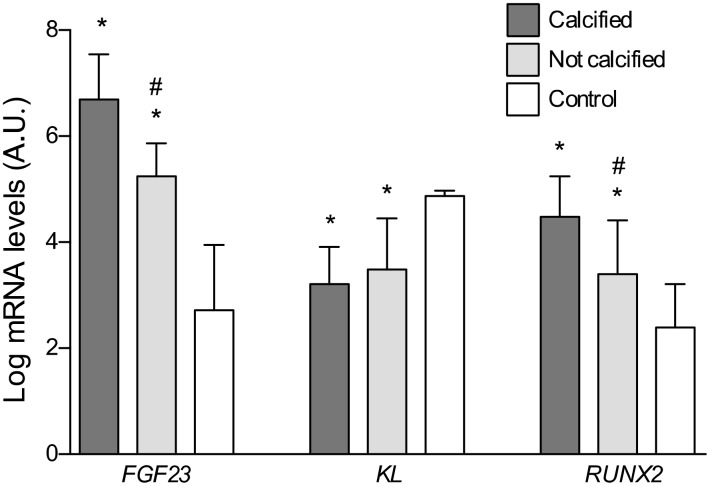
**Differences in the log-transformed gene expression levels of *FGF23, Klotho*, and *RUNX2* attending to the presence of calcification.** *P<0.01 vs. control group; #P<0.01 vs. calcified group.

Fresh sections of the same vascular fragments employed in the histological study were used for the gene expression analysis of *FGF23*, *Klotho* and *RUNX2* genes. The gene expression levels of *FGF23* and *RUNX2* were significantly higher in vascular samples with calcification when comparing to either not calcified samples (P<0.05) or those from subjects in the control group (P<0.01) (Figure 5). A correlation analysis to determine the potential interrelationship among these variables showed that FGF23 expression was direct and significantly associated with *RUNX2* expression levels (r=0.74, P<0.01). Conversely, expression levels of *Klotho* were reduced in both groups of patients with atherosclerotic vascular disease when comparing to control subjects (P<0.01). Gene expression analysis also revealed that *FGF23* expression was significantly higher in the upper tertiles of soluble FGF23 (P<0.05). Conversely, expression levels of Klotho showed a trend to decrease in those tertiles, although the differenced did not reach statistical significance (Table 3).

## DISCUSSION

The main finding of the present study is that patients with clinical atherosclerotic artery disease and VC present significantly higher serum concentrations of FGF23 and increased FGF23 protein immunoreactivity and gene expression levels in the vascular wall as compared with patients without VC. Whether the raise in systemic and vascular levels of FGF23 may directly promote or favor the development and progression of the calcifying process in the vascular beds is currently a matter of debate.

FGF23 is a hormone produced by bone cells which promotes phosphaturia and inhibits the renal production of calcitriol [5]. A recent meta-analysis, collectively comprising more than 20,000 individuals with CKD and subjects with normal renal function, has revealed that higher FGF23 concentrations are associated with greater risks of CV morbidity and mortality [13]. Circulating FGF23 can be found both as intact molecules and C-terminal fragments. Although it is accepted that the full activity is held by the intact form, conflicting results have been reported about the possible activity of the C-terminal fragments, from a maintained activity [14] to an antagonist effect [15]. Moreover, most of the published studies only have measured one of these forms. In the present work, we determined the levels of FGF23 by quantifying the intact protein and the C-terminal peptides, showing that in patients with established clinical atherosclerotic CVD both forms of FGF23 are increased in subjects with VC respect to those without calcification. Similarly, the serum concentrations of any form of soluble FGF23 in the whole group of patients with CVD (including patients with and without VC) are higher than that observed in the control group.

Calcification of arterial vessels, regardless of whether the calcified lesion is generated in the intimal or media layers, is an active process initiated and regulated via a variety of molecular signaling pathways [1, 2, 16]. Different studies have reported an association between higher soluble FGF23 concentrations and abdominal aortic calcification [17], higher coronary calcium scores [18] and coronary artery calcified plaques [19], suggesting a potential pathogenic role of FGF23. However, a causal relationship cannot be determined at present time. Since the higher levels of FGF23 observed in the patients with VC could be explained by the increase in the bone turnover, we determined the levels of TAP. This is the first bone turnover marker to be used in both the clinical and research setting. As expected, we observed that the levels of this protein were higher in the group of patients with VC. However, we did not find increased levels of TAP, nor in Ca or P, in the higher tertiles of FGF23, indicating that the increase in the levels of this phosphatonin is not accompanied with an elevation of TAP levels. However, we cannot rule out that bone regeneration is playing a role in increasing FGF23 levels in these patients. Moreover, in the light of these results it is plausible to suggest that FGF23, a hormone produced by osteoblasts and osteocytes [20], could be also produced in the osteodifferentiated vascular tissue, constituting an extraosseous source of this protein and, therefore, a potential contributor to the circulating levels of FGF23. Nevertheless, we also detected, in a lesser extent, the presence of FGF23 in non-calcified atherosclerotic lesions, indicating that the regulatory mechanisms that governs the synthesis of this hormone at the vascular levels are complex. The presence of FGF23 in these tissues could be due to early stages of the atherosclerotic-derived calcifying process that could not be diagnosed clinically.

FGF23 has been described in calcified atherosclerotic carotid lesions in subjects with normal renal function [12] and in calcified coronary arteries of CKD patients [11]. In this work, we analyzed the expression of FGF23 in calcified lesions from carotid, aorta, and femoral arteries. Our results confirm the presence of FGF23 assessed by immunohistochemistry and gene expression studies. In agreement with the observations by Voigt et al. [12], we noticed intracellular detection of FGF23 which indicates local synthesis of the protein in the vascular wall rather than capture from the circulation. The frequency of samples positive for FGF23 staining was higher in patients with VC as compared to patients without evidence of VC (81.8% vs 57.1%). Additionally, mean immunoreactivity levels for FGF23 were also higher in calcified vessels compared to vascular samples without calcified lesions. Consistent with this, we observed significantly higher gene expression levels of FGF23 in calcified vascular samples than in those without calcification. Interestingly, FGF23 positive staining was also observed in 4 of 18 samples from the control group (aorta and iliac arteries) although all of them showed no staining for calcium deposition. These 4 samples also showed expression of FGF23 gene while the rest of the group did not showed presence of the transcript.

van Venrooij et al. [11] reported a direct relationship between vascular expression of FGF23 and calcium deposition scores in coronary arteries from patients elected for cardiac transplantation. In addition, they found a significant and direct correlation between FGF23 expression levels and the immunoreactivity scores for the osteocyte marker dentin matrix protein (DMP) 1. In our work, we assessed the expression of *RUNX2* gene, a key transcription factor associated with osteoblast differentiation, to consider the relationship between osteoblast lineage markers and vascular expression of FGF23. In the vascular samples from surgery patients, gene expression levels of *RUNX2* were significantly higher in the fragments with calcification as compared to not calcified pieces. Additionally, in the control group we only observed expression of this early osteoblast marker in the samples from the 4 subjects who showed positive staining for FGF23. Considering the absence of calcium deposits and the lower expression of *RUNX2* in these samples as compared with those from surgery patients, this might suggest that the vascular wall was going through the early stages of osteogenic differentiation and, thus, FGF23 expression might be an early feature of the calcification process. Furthermore, we found a significant direct correlation between expression levels of *FGF23* and *RUNX2* genes in all the samples with detectable transcripts, supporting the link between the expression of FGF23 and vascular osteodifferentiation.

The endocrine and autocrine effects of FGF23 on the arterial vessels could be explained partially because of the expression of the main elements of the FGF23/Klotho system in the vascular wall [10]. In our study, FGF23 was co-expressed in the vascular tissue along with FGFR1, FGFR3 and its obligated co-receptor Klotho, suggesting that the vascular tissue is a target for this hormone. Similar to the study of van Venrooij et al. [11], we observed expression of FGFR1 and FGFR3 in calcified plaques but, more important, immunoreactivity levels of both receptors are significantly increased in vascular lesions of patients with established CVD in comparison to the control group.

The expression of Klotho in the vascular tissue, along with its soluble form, has been reported to play potential beneficial effects on the vasculature [21], having been suggested that CVD is a state of vascular Klotho deficiency [22, 23]. According with these results, in the present work the levels of Klotho protein and gene expression are decreased in vessels of the vascular surgery patients group compared to those of the control group. Moreover, immunohistochemical staining enabled the detection of the Klotho transcripts and the intracellular localization of Klotho, which confirms that the presence of this protein is not due to trapping from the blood but it is synthesized by the arterial wall. It is possible that the over-expression of FGFR1 and FGFR3 seen in the vascular wall of patients with vascular disease is a response to remain this tissue sensitive to FGF23 despite the loss of Klotho expression. Interestingly, the similar protein levels of these receptors among patients with or without VC indicate that these elements of the machinery for FGF23 signaling are conserved in the calcified vessels.

Finally, although previous studies have reported the vascular expression of FGF23 and its circulating levels in the setting of VC [12], the relationship between these parameters has not been studied. In this work, we show that vascular immunoreactivity and gene expression levels for FGF23 are higher in the upper tertiles of both soluble forms of FGF23. Therefore, we demonstrate that not only both forms of soluble FGF23 are increased in patients with VC but also that their levels mirror the observed increase in protein and gene expression levels of vascular FGF23. This fact points to the existence of common regulatory mechanisms for the synthesis of this molecule in different organs and tissues. It is likely that the same mechanisms leading to increased circulating levels of FGF23 [24] by stimulating bone gene expression [25] are also present in the arteries of patients who develop VC leading to the increased vascular expression of FGF23. Interestingly, several patients with positive vascular FGF23 staining had no signs of calcification, which might represent an earlier marker for the development of VC before calcium deposits can be evident.

Detection of FGF23 in other tissues different from bone, like vascular calcified walls, suggests that the local expression of this protein might play potential direct roles in the maintaining of the vascular health or in the development of pathological processes. Some authors have already suggested that the expression of FGF23 in calcified plaques could be a local defense mechanism rather than a contributing factor to the mineralization of the vascular wall, considering that FGF23 overexpression *in vitro* suppresses osteoblast differentiation [12, 26]. Moreover, FGF23 indirectly regulates osteopontin secretion in bone matrix by suppressing alkaline phosphatase transcription and phosphate production in osteoblastic cells, acting through FGFR3 in a Kl-independent manner [27] and directly inhibits the osteoblastic Wnt pathway through a soluble Klotho/MAPK-mediated process that required the induction of the Wnt inhibitor Dkk1 [28]. These actions constitute a novel autocrine/paracrine feedback mechanism for the local fine-tuning of bone mineralization at the skeletal level that could be also present at the vascular level. In any case, the biological meaning of this extraosseous expression of FGF23 is a question that still needs to be addressed in mechanistic and functional studies.

It is known that vascular calcification increases arterial stiffness which is associated with low renal tissue oxygenation [29]. Tissue hypoxia is the main stimuli for the expression of the of HIF-1 (hypoxia-inducible factor-1alpha), the key hypoxic transcription factor, which is a main inductor of the osteochondrogenic differentiation of VSMCs [30]. Interestingly, recent *in vitro* studies have demonstrated that HIF-1 mediates FGF23 production in tumor-induced osteomalacia (TIO), a rare paraneoplastic syndrome characterized by the aberrant production of FGF23. Specifically, immunoprecipitation assays confirmed binding of HIF-1 to a consensus sequence within the proximal FGF23 promoter, demonstrating that HIF-1 is a direct transcriptional activator of FGF23 [31]. Therefore, it is plausible to speculate with the existence of a vicious circle by which increased FGF23 may contribute to vascular calcification that results in tissue hypoxia with the stimulation of HIF-1 and the subsequent transcriptional activation of FGF23 production. Finally, data about the relationship between hypoxia and Klotho are scarce. However, a very recent work has shown that patients with obstructive sleep apnea present lower plasma levels of Klotho as compared with healthy control volunteers. Interestingly, a significant association was observed between reduced Klotho concentrations and markers of overnight hypoxemia [32]. Thus, is also possible to consider that tissue hypoxemia induced by vascular calcification may be a contributing factor to low Klotho levels.

Although presenting new interesting data, we acknowledge several limitations in this work. First, serum concentrations of vitamin D and parathyroid hormone -factors related to the FGF23/Klotho system with potential impact on vascular calcification- were not measured and, therefore, their possible influence on the relationship between FGF23 and calcification cannot be completely ruled out. Secondly, the study involved a small group of patients, so the findings may not be generalizable and further studies including larger groups with and without VC are necessary to confirm these observations. Finally, given the cross-sectional design of the analysis, our results show associations, but a causal relationship between FGF23 and calcification, and the role of calcified vessels as an extraosseous source of FGF23 cannot be definitely demonstrated.

In conclusion, our findings link VC with the increased expression of FGF23 in the vessels and with the elevation of circulating levels of the two soluble forms of this hormone. Moreover, the raise in vascular expression of FGF23 occurs along with some of the members of the FGF23/Klotho system supporting the idea that signaling pathways mediating the effects of FGF23 remain active in the calcified tissue. Whether the increase in all these levels is a mechanism that may directly promote the development and progression of the calcifying process or if it constitutes a defense against the lesion is still a matter of debate that needs further investigation.

## Clinical perspectives

1. Vascular calcification constitutes a major risk for cardiovascular mortality. Fibroblast growth factor (FGF) 23 is a phosphaturic osteocyte-derived hormone produced in response to phosphate levels. Epidemiological studies suggest that FGF23 is causally related to cardiovascular mortality and, importantly, that may exert direct effects on cardiovascular system. The synthesis of FGF23 by extra-osseous calcified tissues and its contribution to cardiovascular disease is an intriguing question not adequately studied.
2. This study shows that patients with clinical atherosclerotic artery disease and vascular calcification present significantly higher serum concentrations of FGF23 and increased FGF23 protein immunoreactivity and gene expression levels in the vascular wall as compared with patients without vascular calcifications.
3. These findings provide new mechanistic insights about the pathophysiology of vascular calcification. The interrelationship between FGF23 and calcification in the vascular bed opens new research pathways regarding the development and progression of cardiovascular disease, with evident clinical applicability in prognostic, diagnostic, and therapy.

## MATERIALS AND METHODS

### Patients

One-hundred and eighty patients undergoing an elective open vascular surgery procedure at the Vascular Surgery Service of the University Hospital Nuestra Señora de Candelaria (UHNSC), Santa Cruz de Tenerife, Spain, due to established clinical atherosclerotic artery disease were considered for initial enrollment in this study between November 2014-July 2016. Exclusion criteria included hemodynamic instability; history of chronic inflammatory, immunologic, or tumoral disease; positive serology to hepatitis B, hepatitis C or HIV; acute inflammatory or intercurrent infectious episodes in the previous month; institutionalization; treatment with immunotherapy or immunosuppressive drugs; previous organ transplantation and ESRD. In addition to these patients, 20 cadaveric organ donors without any medical history of CVD were considered as a control group. The study protocol was approved by the Institutional Ethics Committee of the University Hospital Nuestra Señora de Candelaria and complied with ethical standards of the Declaration of Helsinki. Written informed consent was obtained from all participants.

### Samples and biochemical markers

During surgery, a sample of the carotid, aorta, or femoral arteries, according to the affected vessel, was obtained from the participants. From individuals included in the control group, vascular fragments of aorta or iliac arteries were obtained during organ retrieval surgery.

Serum samples were drawn at the same time as the vascular samples, aliquoted, and immediately stored at -80°C until further analysis. Routine biochemical parameters were determined using standard methods. Serum levels of FGF23 were measured by two different enzyme-linked immunosorbent assay (ELISA) kits. Thereby, soluble intact FGF23 (iFGF23) levels were determined by Human FGF23 ELISA Kit (EMD Millipore Corporation, Milford, MA, USA), which detects only the intact form, with a sensitivity of 3.5 pg/mL and intra-and inter-assay coefficients of 9.5% and 6.85%, respectively. For comparative purposes, serum samples from a subgroup of patients with CVD and of all the subjects in the control group were also assayed with the Immutopics Human FGF23 (C-term) ELISA kit (Immutopics Inc., San Clemente, CA), which detects both iFGF23 and C-terminal (cFGF23) fragments, and presents a sensitivity of 1.5 relative units (RU)/mL and intra- and inter-assay coefficients of 1.9% and 3.55%, respectively. Serum levels of Klotho were measured by a solid phase sandwich ELISA (Immuno-Biological Laboratories, Takasaki, Japan), with a sensitivity of 6.15 pg/mL and intra- and inter-assay coefficients of variation of 3.1 % and 6.9 %, respectively.

### Immunohistochemistry and histochemical analysis

Recovered human blood vessels tissues were fixed in 4% buffered formalin for 24 hours. Subsequently, pieces were dehydrated in ascending series of ethanol, cleared in xylene and embedded in paraffin. Blocks were trimmed and 3 μm sections were processed for immunohistochemistry. In brief, sections were deparaffinized in xylene and rehydrated in graded ethanol concentrations. Endogenous peroxidase was blocked with 3% hydrogen peroxide, followed by primary antibody incubation. Primary antibodies used were: rabbit polyclonal anti-FGF23, 1:250 dilution (Bi-Orbit); mouse monoclonal anti-FGFR1, 1:400 dilution (Abcam); rabbit polyclonal anti-FGFR3, 1:300 dilution (Abcam); and rabbit polyclonal anti-Klotho, 1:100 dilution (Abcam). Then, sections were washed three times in PBS and incubated with the Novolink Polymer Detection® (RE7140-K, Novocastra), followed by reaction with 3,3´diaminobenzidine as chromogen. Slides were counterstained with hematoxylin.

For the quantification analysis, a total of 5 images, that include intima and media layers, of each slide were captured and processed with a high-resolution video camera (Sony, DF-W-X710) connected to a light microscope (Nikon Eclipse 50i) using the 4x optical microscope. Then, images were digitalized and the area of tissue stained by the antibodies was quantified by using ImageJ software (Rasband, W.S., ImageJ, National Institutes of Health, Bethesda, MD, USA). Results are expressed in square microns. For confirmation of calcification, sections were also stained with Alizarin red S and von Kossa staining following standard procedures. Images were captured and processed as described above.

### Quantitative real-time PCR

Total RNA was isolated from fresh vascular tissue samples after complete homogenization in TRI Reagent® (Sigma-Aldrich, MO, USA) employing TissueRuptor (Qiagen, Hilden, Germany). Further purification was performed using RNeasy Mini kit (Qiagen), according to manufacturer’s specifications and quantified using a Thermo Scientific NanoDrop Lite Spectrophotometer (Thermo Fisher Scientific, MA, USA). cDNA was obtained using a High Capacity RNA-to-cDNA kit (Applied Biosystems, Foster City, CA, USA) for further analysis by quantitative RT-PCR. Transcripts encoding for *FGF23, Klotho, runt-related transcription* factor 2 (*RUNX2*), and *glyceraldehyde 3-phosphate dehydrogenase* (*GAPDH*) were measured by TaqMan real-time quantitative PCR (qRT-PCR) with TaqMan Fast Universal PCR Master Mix (Thermo Fisher Scientific). TaqMan gene expression assays for each transcript (Hs00221003_m1 [FGF23], Hs00183100_m1 [KLOTHO], Hs00231692_m1 [RUNX2], Hs99999905_ m1 [GAPDH]) were analyzed in a 7500 Fast Real-Time PCR System (Thermo Fisher Scientific). The level of target mRNA was estimated by relative quantification using the comparative method (2^-ΔΔCt^) by normalizing to *GAPDH* expression. mRNA levels were expressed as arbitrary units (a.u.). Quantification of each cDNA sample was tested in triplicate. A corresponding non-reverse transcriptase reaction was included as a control for DNA contamination.

### Statistical analysis

All analyses were performed using SPSS software version 25 (IBM Corp. Armonk, NY, USA). Continuous variables are reported as mean±SD or median with interquartile range (IQR) as appropriate, and categorical data as number and percent frequency. Continuous variables were checked for the normal distribution assumption using the Kolmogorov–Smirnov statistics, and those that did not satisfy the criteria were log-transformed to attain normal distribution. Spearman correlation coefficient was calculated to assess association between variables. Comparisons between groups were performed by Mann–Whitney U or Chi-square test. A two tailed P value <0.05 was considered statistically significant.
